# Synthesis of umbelliferone derivatives in *Escherichia coli* and their biological activities

**DOI:** 10.1186/s13036-017-0056-5

**Published:** 2017-04-05

**Authors:** Luan Luong Chu, Ramesh Prasad Pandey, Haet Nim Lim, Hye Jin Jung, Nguyen Huy Thuan, Tae-Su Kim, Jae Kyung Sohng

**Affiliations:** 1 Department of Life Science and Biochemical Engineering, Sun Moon University, 70 Sunmoon-ro 221, Tangjeong-myeon, Asan-si, Chungnam 31460 Republic of Korea; 2 Department of BT-Convergent Pharmaceutical Engineering, Sun Moon University, 70 Sunmoon-ro 221, Tangjeong-myeon, Asan-si, Chungnam 31460 Republic of Korea; 3 Center for Molecular Biology, Institute of Research and Development, Duy Tan University, K7/25 Quang Trung, Danang, Vietnam

**Keywords:** Glycosylation, Hydroxylation, Methylation, Umbelliferone

## Abstract

**Background:**

Umbelliferone, also known as 7-hydroxycoumarin, is a phenolic metabolite found in many familiar plants. Its derivatives have been shown to have various pharmacological and chemo-preventive effects on human health. A uridine diphosphate glycosyltransferase YjiC from *Bacillus licheniformis* DSM 13, a cytochrome P450BM3 (CYP450 BM3) variant namely mutant 13 (M13) from *Bacillus megaterium*, and an *O*-methyltransferase from *Streptomyces avermitilis* (SaOMT2) were used for modifications of umbelliferone.

**Results:**

Three umbelliferone derivatives (esculetin, skimmin, and herniarin) were generated through enzymatic and whole cell catalysis. To improve the efficiencies of biotransformation, different media, incubation time and concentration of substrate were optimized and the production was scaled up using a 3-L fermentor. The maximum yields of esculetin, skimmin, and herniarin were 337.10 μM (67.62%), 995.43 μM (99.54%), and 37.13 μM (37.13%), respectively. The water solubility of esculetin and skimmin were 1.28-folds and 3.98-folds as high as umbelliferone, respectively, whereas herniarin was 1.89-folds less soluble than umbelliferone. Moreover, the antibacterial and anticancer activities of herniarin showed higher than umbelliferone, esculetin and skimmin.

**Conclusions:**

This study proves that both native and engineered enzymes could be employed for the production of precious compounds via whole cell biocatalysis. We successfully produced three molecules herniarin, esculetin and skimmin in practical amounts and their antibacterial and anticancer properties were accessed. One of the newly synthesized molecules the present research suggests that the combinatorial biosynthesis of different biosynthetic enzymes could rapidly promote to a novel secondary metabolite.

**Electronic supplementary material:**

The online version of this article (doi:10.1186/s13036-017-0056-5) contains supplementary material, which is available to authorized users.

## Background

**Fig. 1:**
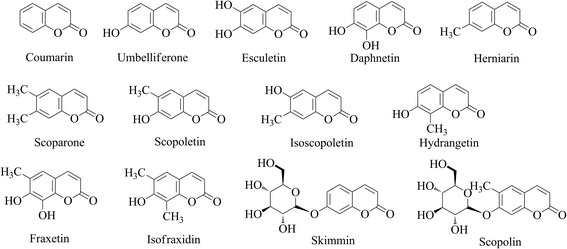
The chemical structure of the simple coumarins

Umbelliferone and its derivatives are compounds derived from coumarin (Fig. 1) with pharmacological and chemopreventive benefits for human health. Umbelliferone is known to have antinociceptive and anti-inflammatory activities in animal models [1]. Moreover, esculetin exhibits various biological activities including antioxidant [2], anti-tumor, anti-metastatic [3], anti-proliferative, pro-apoptotic [4], and neuroprotective activities [5]. Umbelliferone glycosides have been found to possess neuroprotective effects against serum deprivation-induced PC12 cell damage [6] with antidiabetic, antihyperlipidemic, and antioxidative activities [7]. Furthermore, 7-methoxycoumarin showed antifugal and antibacterial activities [8]. Likewise, other umbelliferone derivatives such as scopoletin, scoparone, fraxetin, esculin, and daphnetin have been reported to have antioxidant and intestinal anti-inflammatory activities [9].

Coumarins are phenolic metabolites commonly distributed in many plant species [10]. The *ortho*-hydroxylation of cinnamate is a pivotal step in the biosynthesis of coumarin in plants. The core structure, 2*H*-1-benzopyran-2-one, of umbelliferone and its derivatives are formed via *ortho*-hydroxylation of cinnamates which undergo *trans/cis* isomerization of the side-chain followed by lactonization [11]. Although these compounds exhibit multi-beneficial pharmacological properties, isolation and purification of umbelliferone and its derivatives from plants are problematic due to low concentration and seasonal and regional dependency [12]. On the other hand, chemical synthesis requires the usage of hazardous agents, long synthetic steps, and extreme reaction conditions [13, 14].

Recently, an alternative approach has emerged as a promising method to synthesize small molecules by engineering microbes. Simple coumarins have been obtained from engineered *Escherichia coli* (*E. coli*) by combinational expression of artificial pathway enzymes with diverse genetic source including phenylalanine ammonia lyase (PAL) or tyrosine ammonia lyase (TAL), 4-cinnamic acid:coenzyme A ligase (4CL), coumarin synthase (C2´H, coumaroyl-CoA 2′-hydroxylase; or F6´H, feruloyl-CoA 6′-hydroxylase) [15–17]. For example, umbelliferone and scopoletin have been synthesized by using TAL, HpaBC (4-hydroxyphenylacetate 3-hydroxylase), and CCoAOMT (caffeoyl-CoA *O*-methyltransferase) in conjunction with 4CL and F6´H from *p*-coumaric acid and ferulic acid, respectively [16]. Moreover, tyrosine can be converted to esculetin by employing TAL, C3H (coumarate 3-hydroxylase from *E.coli*) or Sam5 (a monooxygenase from *Saccharothrix espanaensis*), 4CL, and F6´H [17, 18]. These researches demonstrated that hydroxyl cinnamic acid or glucose was the strating material for biosynthesis of simple coumarins. However, low catalytic activity, low final yield, and limited products are the most frequently encountered problems when applying a combinatorial expression approach.

In this study, biocatalyst system was carried out to modify umbelliferone by using glycosyltransferase, cytochrome P450, and *O*-methyltransferase. Three derivatives of umbelliferone, namely esculetin, skimmin, and herniarin, were successfully synthesized and their antibacterial and anticancer activities were evaluated.

## Results

### Protein expression and purification

Recombinant proteins CYP450 BM3 (119 kDa), M13 (119 kDa), YjiC (45 kDa), and SaOMT2 (37.5 kDa) were overexpressed in *E. coli* BL21 (DE3) (Additional file 1: Figure S1). The content acquired from the soluble fractions of proteins CYP450 BM3 and M13 were 477.253 and 1932.069 nM/L, respectively. The concentrations of crude proteins of CYP450 BM3, M13, YjiC, and SaOMT2 were determined to be approximately 147.54, 122.93, 344.28, and 76.99 μg/mL, respectively, based on the Bradford method.

### Enzymatic reaction

**Fig. 2:**
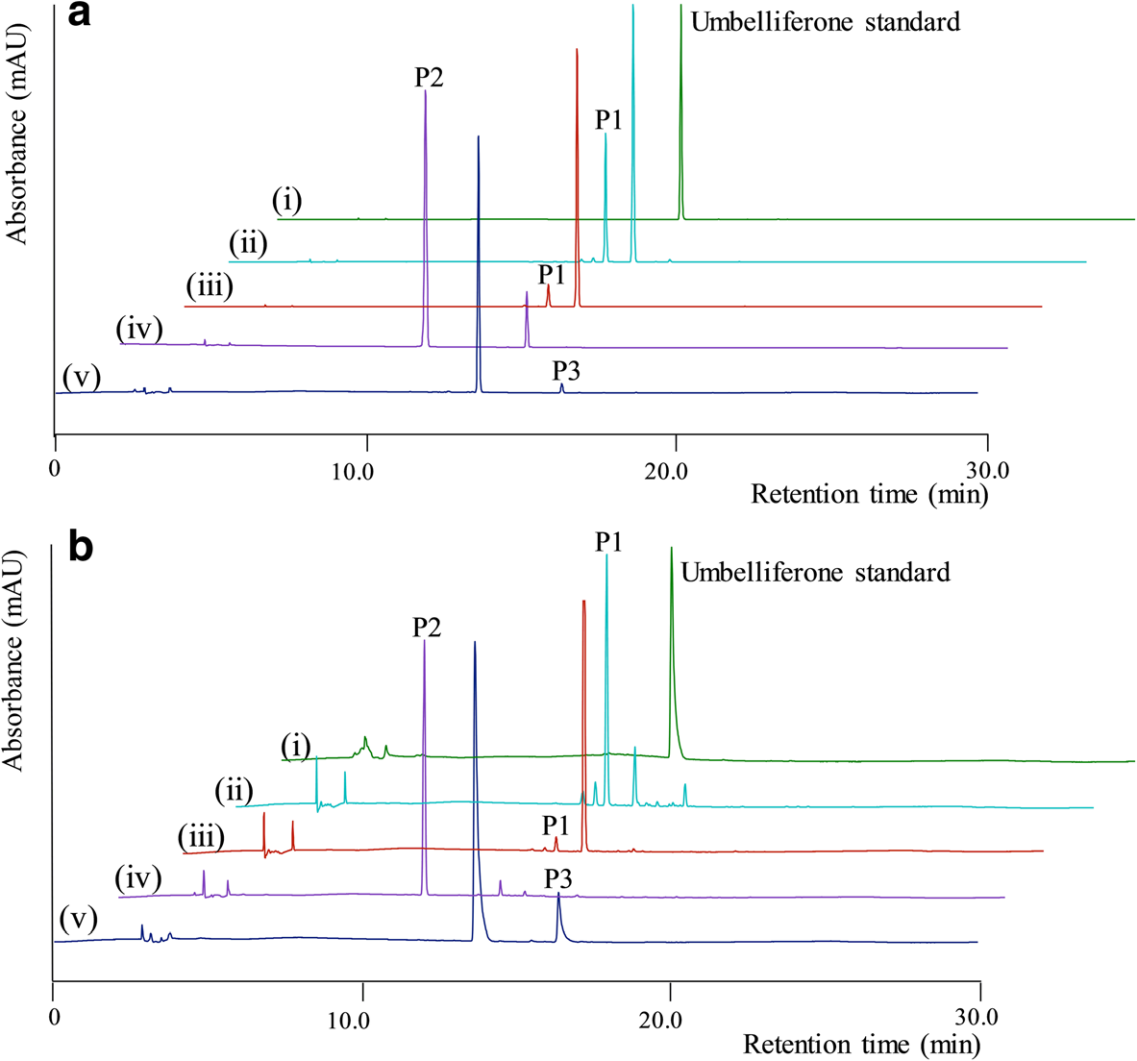
**a** In vitro reaction mixture and **b** whole cells bioconversion of umbelliferone on the HPLC-PDA analysis. *(i*) control reaction of umbelliferone using *E. coli* BL21 (DE3); (*ii*) hydroxylation with M13 and (*iii*) CYP450 BM3 at 48 h; (*iv*) glycosylation with YjiC at 12 h; and (*v*) methylation with SaOMT2 at 48 h, respectively

The four enzymes was performed enzymatic reaction using umbelliferone as standard substrate. The results were analyzed by high performance liquid chromatography-photodiode array (HPLC-PDA) and high-resolution quadruple time-of-flight electrospray ionization-mass spectrometry (HR-QTOF ESI/MS). The retention time (*t*_R_) of umbelliferone standard was observed at 13.749 min with UV absorbance at 330 nm (Fig. 2a(i)). The found mass of umbelliferone was ~ 163.0394 [M + H]^+^ *m/z* ^+^ equivalent to molecular formula C_9_H_7_O_3_, for which the calculated mass was 163.0395 (Additional file 1: Figure S2A). HPLC-PDA chromatogram revealed the appearance of a new peak (P1) in the hydroxylation reaction of both CYP450 BM3 and M13. A new peak *t*_R_ at 12.730 min (Fig. 2a(ii;iii)) and *λ*_max_ at 325 nm might be hydroxylated umbelliferone. P1 was further analyzed by HR-QTOF ESI/MS. The mass spectrum displayed a peak with found mass of ∼ 179.0390 [M + H]^+^ *m/z* ^*+*^, resembling the mass of mono-hydroxylated derivative of umbelliferone with a molecular formula C_9_H_7_O_4_, for which the calculated mass was ∼ 179.0344 (Additional file 1: Figure S2B). HPLC-PDA analysis showed that M13 had higher catalytic activity (35.48%) as a monooxygenase than CYP450 BM3 in which the conversion was limited to 8.29% (Table 1, Fig. 2a(iii)). Moreover, the HPLC-PDA analysis of glycosylation reaction mixture showed a new peak at *t*_R_ of 10.323 min (P2) (Fig. 2a(iv)). P2 exhibited the found mass [M + H]^+^ *m/z* ^+^ at ~ 325.0912 with λ_max_:317 nm, corresponding to the calculated mass of the mono-glucoside derivative of umbelliferone with molecular formula C_15_H_17_O_8_ for [M + H]^+^ *m/z* ^+^ ~ 325.0923 (Additional file 1: Figure S2C). Furthermore, the HPLC-PDA analysis of methylation reaction mixture exhibited an additional peak at at *t*_R_ of 16.381 min (P3) (Fig. 2a(v)). P3 showed the exact mass [M + H]^+^ *m/z* ^+^ at ~ 177.0553 with λ_max_:321 nm, corresponding to the calculated mass of methylated umbelliferone with molecular formula C_10_H_9_O_3_ for [M + H]^+^ *m/z* ^+^ ~ 177.0552 (Additional file 1: Figure S2D). The result displayed the conversion of umbelliferone to its derivatives were 86.45% and 2.08% in the glycosylation and methylation reactions, respectively (Table 1). These in vitro results indicated that umbelliferone might be a substrate of various transferase enzymes. It was converted more efficiently to glycosylated form than other derivatives. Therefore, umbelliferone was used for the production of its derivatives by using M13, YjiC, and SaOMT2 in whole cells.

**Table 1:** The HPLC-PDA, chemical formula, HR-QTOF ESI/MS, UV maxima and conversion product analyses of umbelliferone in in vitro reaction using CYP450 BM3 and its variant proteins M13, YjiC and SaOMT2

Name		HPLC (*t*_*R*_) min	Chemical formula	Calculated mass [M + H]^+^ *m/z* ^+^~	Exact mass [M + H]^+^ *m/z* ^+^~	UV maxima (nm)	% Conversion
Substrate	Umbelliferone	13.749	C_9_H_6_O_3_	163.0395	163.0394	327	
Products	Hydroxylated (P1) by P450 BM3	12.730	C_9_H_6_O_4_	179.0344	179.0390	325	8.09
	Hydroxylated (P1) by M13		C_9_H_6_O_4_				35.48
	Glycosylated (P2)	10.323	C_15_H_16_O_8_	325.0923	325.0912	317	86.45
	Methylated (P3)	16.381	C_10_H_8_O_3_	177.0344	177.0547	321	2.08

### Bioconversion of umbelliferone using CYP450 BM3 and its variant

The hydroxylated derivative was produced from umbelliferone by using *E. coli* harboring pCW(Ori^+^)-CYP450 BM3 wild type and *E. coli* harboring pCW(Ori^+^)-mutant 13 (M13). The cells were induced with IPTG and were incubated at 28 °C with 100 μM umbelliferone for 12 h in various media as described in materials and methods. Culture media and cell pellets were extracted with ethyl acetate (EtOAc) and the products were analyzed by HPLC-PDA. HPLC-PDA chromatograms of both strains showed a new peak at *t*_*R*_ ~ 12.730 min (P1) in comparison with umbelliferone standard at *t*_*R*_ ~ 13.749 min under UV absorbance of 330 nm (Fig. 2b(i; ii)). All detected peaks were further analyzed by HR-QTOF ESI/MS. The found mass of umbelliferone standard was observed at ∼ 163.0394 [M + H]^+^ *m/z* ^*+*^ corresponding to molecular formula C_9_H_7_O_3_ with λ_max_ ∼ 327 nm, for which the calculated mass was ∼ 163.0395 (Additional file 1: Figure S2A). The found mass of hydroxylated product P1 at ∼ 179.0390 [M + H]^+^ *m/z* ^*+*^ corresponding to molecular formula C_9_H_7_O_4_ with λ_max_ ∼ 325 nm, for which the calculated mass was ∼ 179.0344 (Additional file 1: Figure S2B).

**Fig. 3:**
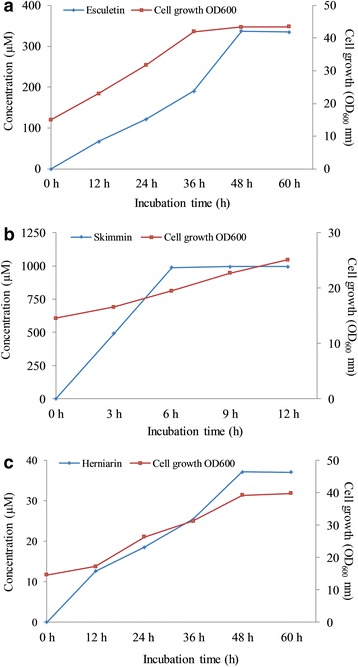
The scale-up of **a** hydroxylated **b** glucosylated **c** methylated umbelliferone and cell growth at OD_600_ nm in 3-L fermentation at different time intervals

The bioconversion rate of umbelliferone to hydroxylated products was very low with the wild type strain using all three different media. However, the conversion was relatively higher with *E. coli* BL21(DE3) harboring pCW(Ori^+^)-mutant 13 (M13). The highest hydroxylated product was recorded to be 84.70 μM (84.7% conversion) in M9 medium, which was 1.85-fold higher than that in Luria-Bertani (LB) medium (45.72 μM) and 2.07-fold higher than that in Terrific Broth (TB) medium (40.84 μM) (Additional file 1: Figure S3A). These results further mean that M9 medium was a suitable medium for the production of hydroxylated product from umbelliferone. Similar trend has been observed with other flavonoid molecules [19]. However, the exact reason behind this is unclear. After that, *E. coli* BL21(DE3) harboring pCW(Ori^+^)-mutant 13 was chosen to optimize the substrate conversion with separately supplied 100, 250, 500, 750, and 1000 μM of umbelliferone. Substrate utilization and cell growth were measured at 12 h intervals. The maximum concentration of hydroxylated product was 329.29 μM (65.86%) at 48 h with OD_600_ ~ 3.949 when 500 μM of umbelliferone was added into the biotransformation reaction. Even though umbelliferone was non-toxic to cells (Additional file 1: Figure S3B), bioconversion was inhibited when a higher amount of substrate was supplied (Additional file 1: Figure S3C). Recombinant strain *E. coli* BL21(DE3) harboring pCW(Ori^+^)-mutant 13 were cultured in a 3-L fermentor with 1 × M9 minimal medium supplemented with 500 μM umbelliferone (~243.2 mg in 3-L) and supplied 2% glucose. The temperature and pH of the fermentor were kept constant at 28 °C and 7.6, respectively. The samples were taken at 12 h interval and measured by HPLC-PDA. Only 377.0959 μM (~180.2 mg; 60.1 mg/L) of hydroxylated umbelliferone was produced until 48 h as the maximal yield with bioconversion rate of the substrate at OD_600_ nm ~ 43.37 of approximately 67.62% (Fig. 3a).

### Production of glucosylated umbelliferone

The glucosylated derivative was synthesized from umbelliferone by using *E.coli* harboring pET28a-YjiC. This *E. coli* strain was fed with 100 μM substrate at 20 °C for 12 h in various media. The product was extracted with twice volume of EtOAc. HPLC-PDA analysis showed a new peak (P2) with *t*_R_ ~ 10.323 min compared to *t*_R_ ~ 13.749 min of a substrate under UV absorbance of 330 nm (Fig. 2b(i; iii)). The found mass of glycosylated product P2 [M + H]^+^ *m/z* ^*+*^ was ∼ 325.0912, resembling molecular formula C_15_H1_7_O_8_ with λ_max_ ∼ 317 nm. The calculated mass was ∼ 325.0923 (Additional file 1: Figure S2C). While the highest bioconversion of umbelliferone to glucosylated derivative was observed in M9 medium (99.65 μM, 99.65%), which was lower in both LB (58.70 μM, 58.70%) and TB (46.91 μM, 46.91%) media under identical reaction conditions. (Additional file 1: Figure S4A). The different concentration of umbelliferone (100, 500, 1000, 1500 and 2000 μM) was fed into the culture of *E.coli* harboring pET28a-YjiC in the M9 1x medium. An equal volume of sample was taken and analyzed at 3 h intervals. Time and concentration dependent study showed that the highest concentration of umbelliferone glucoside was 996.72 μM (99.67%) with OD_600_ ~ 3.06 at 12 h when 1000 μM of substrate was supplemented in the culture. Umbelliferone failed to affect the growth rate of cells (Additional file 1: Figure S4B). However, the amount of glycosylated product was not significantly increased when a higher amount of substrate was supplied (Additional file 1: Figure S4C). Therefore, 1000 μM umbelliferone and 1x M9 minimal medium with 2% glucose were selected for the scale up of bioconversion reaction in 3-L fermentor. Nearly all umbelliferone was transformed into glycosylated product at 6 h, yielding approximately 995.43 μM (~967.6 mg; 322.8 mg/L) of product with OD_600_ nm ~ 22.7 (Fig. 3b).

### Whole cell methylation of umbelliferone

*E. coli* harboring pRSF-Duet-SaOMT2 was subjected to methylation process of umbelliferone. The biotransformation system was supplied with 100 μM and incubated at 20 °C for 48 h in various media. The product was extracted with EtOAc and analyzed by HPLC-PDA. The result showed methylation product of substrate with a peak at *t*_R_ ~ 16.381 min whereas the aglycone moiety of umbelliferone appeared at *t*_R_ ~ 13.749 min (Fig. 2b(iv)). Subsequently, the peak at *t*_R_ ~ 16.381 min was further analyzed by HR-QTOF ESI/MS. The observed mass [M + H]^+^ *m/z* ^*+*^ was ∼ 177.0553, resembling molecular formula C_10_H1_9_O_3_ with λ_max_ ∼ 321 nm. The calculated mass of methylated umbelliferone was ∼ 177.0552 (Additional file 1: Figure S2D).

Unlike hydroxylation and glycosylation reactions in whole cell biotransformation, methylated product was produced at maximum concentration (17.61 μM, 17.61% conversion) after 48 h incubation in LB media, followed by that in M9 (2.60 μM, 2.6%) and TB media (1.15 μM, 1.15%) (Additional file 1: Figure S5A). The results of optimized conversion showed that the maximum concentration of methylated product was 17.61 μM (17.61%) at 48 h with OD_600_ ~ 3.45 when 100 μM of umbelliferone was added into the biotransformation reaction (Additional file 1: Figure S5B). Umbelliferone did not affect the growth rate of cells. However, the bioconversion from a substrate to the methylated product was not meaningfully increased when a higher amount of substrate was supplied (Additional file 1: Figure S5C). To test the reproducibility of the bioconversion process into the large-scale fermentor, batch fermentation of this strain was carried out in a 3-L fermentor with LB medium supplied with umbelliferone at 100 μM (~48.64 mg in 3-L). The temperature and pH of the fermentor were maintained at 25 °C and 7.6, respectively. The samples were detached at 12 h interval and measured by HPLC-PDA. HPLC-PDA analysis revealed that methylated product bioconversion was increased to 37.13 μM (~19.6 mg; 6.5 mg/L) at 48 h with OD_600_ nm ~ 39.21 (Fig. 3c).

### Structural elucidation of umbelliferone and its derivatives

The structures of umbelliferone standard and purified modified products were analyzed by proton nuclear magnetic resonance (^1^H-NMR) and carbon-13 nuclear magnetic resonance (^13^C-NMR) at 700 MHz in dimethyl sulfoxide-d_6_ (DMSO-*d*_6_). We re-confirmed umbelliferone based on the previous report (Additional file 1: Figure S6) [20]. The ^1^H-NMR spectrum of hydroxylated umbelliferone showed the absence of proton signal at *δ* = 6.79 ppm (*J* = 8.4, 2.3 Hz) for C-6. It showed the presence of a new signal at *δ* = 10.09 ppm (s) for a hydroxyl group. An upfield shift at *δ* = 139.02 ppm of the C-6 of hydroxylated product was also observed in comparison with the same carbon of umbelliferone at *δ* = 113.59 ppm accompanied with a downfield shift of the resonances of adjacent carbons C-7 at *δ* = 158.21 ppm and *δ* = 160.92 ppm, respectively (Table 2; Additional file 1: Figure S7). Furthermore, the compound was identical to an authentic sample of esculetin, a hydroxylated derivative of umbelliferone [21].

**Table 2:** Umbelliferone in comparision with hydroxylated (esculetin), glucosylated (skimmin) and methylated umbelliferone (herniarin) on ^1^H-NMR and ^13^C-NMR analyses

Carbon No.	Umbelliferone		Esculetin		Skimmin		Herniarin	
	^1^H NMR	^13^C NMR	^1^H NMR	^13^C NMR	^1^H NMR	^13^C NMR	^1^H NMR	^13^C NMR
2		160.92		158.21		158.64		160.77
3	6.20 (d, J = 9.4 Hz).	111.87	6.70 (d, *J* = 2.3 Hz)	113.61	6.33 (d, *J* = 9.5 Hz)	113.75	6.30 (d, *J* = 9.4 Hz)	112.89
4	7.93 (d, J = 9.4 Hz)	145.00	7.35 (d, *J* = 8.4 Hz)	127.73	8.01 (d, J = 9.5 Hz)	144.74	8.00 (d, *J* = 9.5 Hz)	144.84
4a		111.75		112.72		113.61		101.19
5	7.52 (s)	130.18	7.06 (s)	116.69	7.65 (d, J = 8.6 Hz)	129.91	7.64 (d, *J* = 8.5 Hz)	129.97
6	6.79 (dd, J = 8.4, 2.3 Hz)	113.59		139.02	7.05 (d, *J* = 2.3 Hz)	114.13	6.96 (dd, J = 8.6, 2.4 Hz)	112.96
7		161.76		159.16		160.68		162.96
8	6.72 (s)	102.63	6.74 (dd, *J* = 8.5, 2.3 Hz)	102.36	7.02 (dd, *J* = 8.6, 2.3 Hz)	103.63	7.01 (d, J = 2.4 Hz)	75.85
8a		155.97		151.00		155.50		155.91
1’					5.03 (d, *J* = 7.5 Hz)	100.43		56.42
2’					3.44 (dd, *J* = 10.9, 6.1 Hz)	73.52		
3’					3.38 – 3.22 (m)	77.57		
4’					3.13 (s)	70.03		
5’					3.68 (dd, J = 2.9, 1.1 Hz)	76.85		
6’					4.28 (d, J = 9.1 Hz)	61.05		
Hydroxyl group								
7-OH	10.59 (s)		9.84 (s)					
6-OH			10.09 (s)					
Methyl group								
7-OCH_3_							3.86 (s)	

s singlet, d doublet, dd doublet of doublet, m multiplet

In addition, ^1^H-NMR study of the purified glycosylated product displayed the presence of an anomeric proton at *δ* = 5.03 ppm (d, *J* = 7.5 Hz, H-1´) with a beta (*β*) configuration of the sugar moiety, whereas the sugar region observed between *δ* of 3.13 and 4.28 ppm. In ^13^C-NMR, 6 carbon signals of the *O*-glucoside moiety were found at *δ* = 100.43 (C-1´), *δ* = 73.52 (C-2´), *δ* = 77.57 (C-3´), *δ* = 70.03 (C-4´), *δ* = 76.85 (C-5´), and *δ* = 61.05 ppm (C-6´) (Table 2; Additional file 1: Figure S8A&B). The site of glycosylation at C-7 was confirmed by a correlation between the anomeric proton H-1’ at *δ* = 5.03 ppm and carbon C-7 at *δ* = 160.68 ppm of umbelliferone in Heteronuclear multiple-bond correlation spectroscopy (HMBC) analysis (Additional file 1: Figure S8C). These analyses identified that the glycosylated product was 7-*O-β*-D glucopyranosyl umbelliferone, also known as skimmin [22].

Moreover, the ^1^H-NMR spectrum of the purified methylated product showed a set of signal at *δ* = 3.86 ppm (s, 3H) corresponding to a methyl group. Furthermore, the ^13^C-NMR showed ten carbons signals, including one new methyl group at *δ* = 56.42 ppm representing O-CH_3_ group in comparison with the nine carbon signals of umbelliferone (Table 2; Additional file 1: Figure S9A&B). The site of methylation at C-7 was confirmed by a correlation between methyl group and carbon C-7 at *δ* = 162.95 ppm of umbelliferone in HMBC analysis (Additional file 1: Figure S9C). These data confirmed that methylated product was 7-methoxycoumarin, also known as herniarin [21].

### Water solubility determination

The data showed that the water solubility of herniarin was decreased by 1.89-folds, whereas esculetin and skimmin were significantly improved by 1.28 and 3.98-folds, respectively, compared to that of umbelliferone. These results indicated that hydroxylation and glycosylation of umbelliferone enhanced its water solubility by attachment of hydrophilic moieties, while methylation decreased its water solubility by attachment of hydrophobic methyl moiety.

### Antibacterial activities

Results of disc diffusion assays showed that umbelliferone, esculetin, and skimmin did not exhibit any antibacterial activity against the tested five different human pathogens when 10 μL of 100 mM compound was applied. However, herniarin exhibited antibacterial activity against Gram-positive bacteria *Staphylococcus aureus* subsp. *aureus* KCTC 1916 (*S. aureus*) and *Bacillus subtilis* KACC 17047 (*B. subtilis*) with a zone of inhibition values of 9 ± 0.12 mm and 8.5 ± 0.18 mm, respectively (Additional file 1: Table S1). These results revealed that methylation of umbelliferone at hydroxyl group of the C-7 position might be profitable for heightening its antibacterial activity against Gram-positive bacteria.

### Anticancer activities

**Fig. 4:**
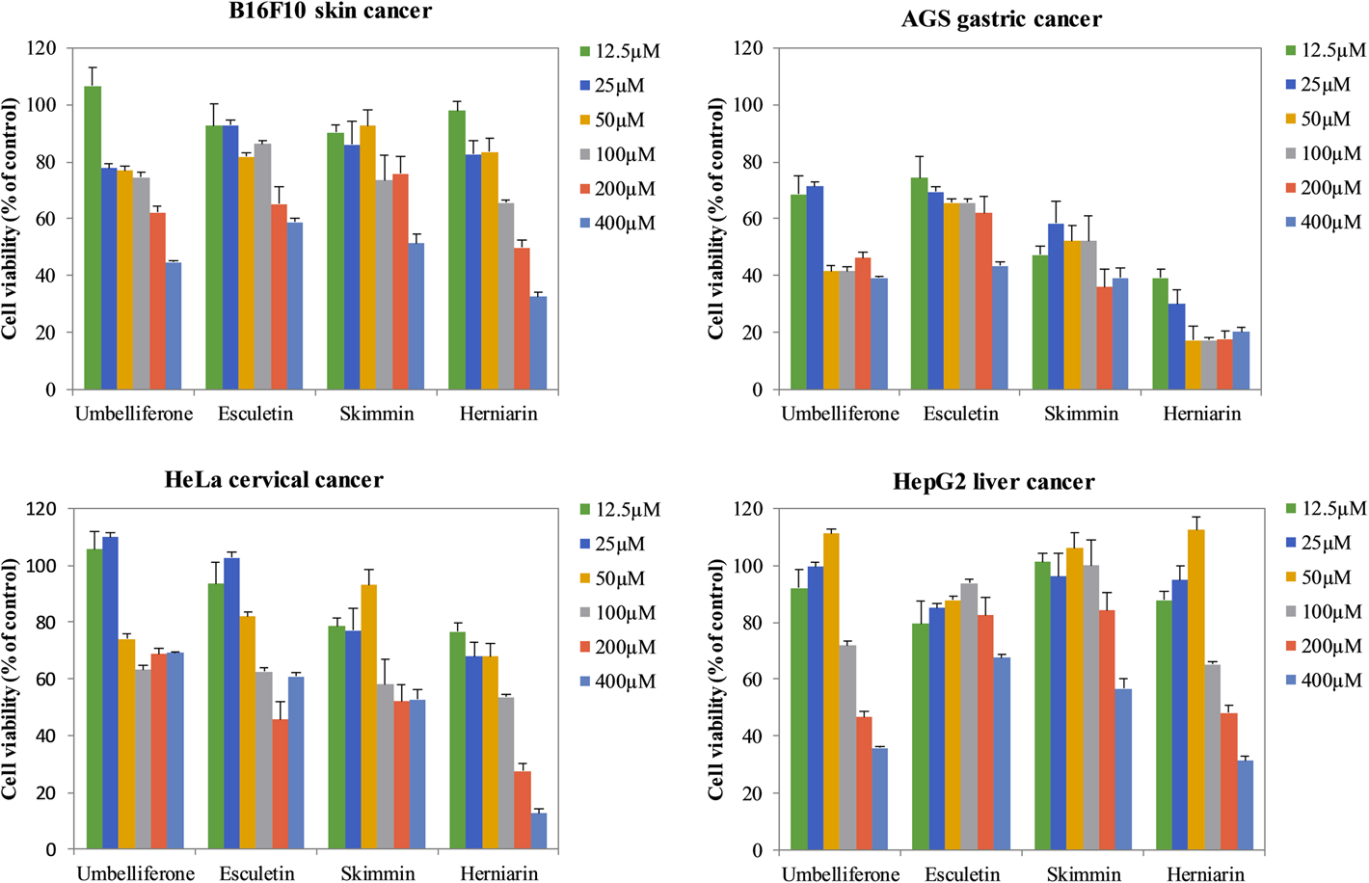
The growth of four cancer cell lines were treated with umbelliferone and its derivatives

All prepared compounds were further evaluated for their in vitro cytotoxicity by 3-(4,5-dimethylthiazol-2-yl)-2,5-diphenyltetrazolium bromide (MTT) colorimetric assay against four different cancer cell lines (Fig. 4). Results showed that herniarin exhibited good cytotoxic activities compared to the other three compounds. Cell viability of skin melanoma (B16F10), gastric carcinoma (AGS), epitheliod cervix carcinoma (HeLa) and hepatic carcinoma (HepG2) reduced approximately 32.63%, 20.25%, 12.79%, and 31.28% (*p <* 0.05), respectively, compared to controls, when treated 400 μM of herniarin. The 50% inhibitory concentration (IC_50_) values of herniarin for B16F10, AGS, HeLa and HepG2 cells were 197.0, 28.29, 80.21, and 206.1 μM, respectively. Skimmin inhibited AGS cell lines with an IC_50_ value of 34.42 μM. However, esculetin did not show any activity against the tested cell lines, whereas umbelliferone exhibited effective anticancer activity against AGS and HepG2 cell lines with IC_50_ values of 129.9 and 222.3 μM, respectively (Additional file 1: Table S2). These results suggest that herniarin and skimmin can remarkable reduce the cell viability of AGS cell line in a dose-dependent manner. This is the first report of the activity of the two compounds against AGS cell line.

## Discussion

Post-modifications of natural compounds is one of the approaches to enhance the biological and pharmaceutical properties of parent molecules [23]. In this research, we produced three umbelliferone derivatives using a monooxygenase, a glycosyltransferase, and a methyltransferase enzyme *via* microbial biotransformation in *E. coli*. First, umbelliferone was oxidized by CYP450 BM3 from *B. megaterium* and its variant M13 to produce metabolite esculetin. Second, a flexible glycosyltransferase YjiC from *B. licheniformis* capable of accepting various small molecules such as chalcone [24, 25], flavonoids [26], stilbene [27], and anthracycline antibiotic were used for efficient glycosylation of umbelliferone to produce skimmin [28]. Finally, we extended acceptor substrate of SaOMT2 from *S. avermitilis* [29] to produce herniarin. Our results suggest that modification of umbelliferone with different enzymes can be used to develop new compounds with novel biological activities.

Enzymatic methods were considerable contributed to the industrial production of pharmaceutical substances [30]. However, there are several shortcomings when utilizing an in vitro enzymatic synthesis approach. Most biocatalysts are limited by cofactor-dependent enzymes such as CYP BM3 which is NADPH-dependent cytochrome P450 [31] and YjiC, a UDP-dependent glycosyltransferase. In addition, NADPH, UDP-glucose, and S-adenosyl-L-methionine (SAM) not only are high cost but also unstable to limit their apply in small scale stoichiometric reaction [32]. Although alternative cofactors-generating systems have been developed for large-scale reactions, the cost of reaction is still too expensive and reaction yield is deficient [33]. For example, only 8.09% and 35.48% of conversion rates from umbelliferone to esculetin were found in in vitro reactions with CYP450 BM3 and M13, respectively. Similarly, methylation reaction of SaOMT2 are limited convert substrate to herniarin (Table 1). Therefore, metabolic engineering and microbial biotransformation approaches are needed to further enhance the production by utilizing indigenous co-factors, ultimately lowering the cost of target molecule production.

Interestingly, the catalytic efficiency of M13 with umbelliferone was 4.39-folds higher than CYP450 BM3 in in vitro reaction. In addition, protein M13 showed higher catalytic efficiency for umbellifferone than wild-type CYP450 BM3 in whole cells for 48 h under the same condition (Table 2; Additional file 1: Figure S3). The long-distance between the heme iron at ferric resting state in the CYP450 BM3 and oxidizable carbon of umbelliferone is one of the reasons for the low bioconversion efficiency [34]. Furthermore, substitution of several key amino acids in the wild-type protein can affect on the activity and selectivity of the biocatalyst reaction. For example, arginine residues at position 47 is supposed to be noticeable for entering of the substrate to the channel and controlling substrate accessibility to the binding pocket [35]. The leucine residues at position 86 could be effective for conformational changes of amino acids near heme, resulting in alteration of the electron transfer pathway to increase activity [36]. The substitution of phenylalanine 87 can directly interact with bound substrates and affect the activity and stereo-selectivity or regioselectivity of enzymes [37]. It is possible that the substituted key amino acid in the wild type protein might have altered CO difference spectra of M13 in comparison with CYP450 BM3. These data indicate that protein engineering was powerful method for enhance the production of natural molecules. They also suggest that one way to improve the activity of SaOMT2 toward umbelliferone is by engineering *via* site-saturation mutagenesis based on the structural model of substrate binding site of the enzyme. Moreover, engineering of YjiC could advance metabolic engineering by tailoring compounds with high stereo-selectivity or regio-selectivity to achieve high yields of desired molecules [38].

We also studied the biological activities of umbelliferone and its three derivatives as such antibacterial and anticancer agents. Although the activities of four compounds were tested against five bacteria, only herniarin was effective against two Gram-positive bacteria *B. subtilis* and *S. aureus*. This indicated that the methyl substitution at the hydroxyl group of the C-7 position reduced the water solubility and showed significant antibacterial activity. The possible reason could explain that the enhanced lipophilicity of alkyl group leads to improving the ability of herniarin to overcome cell membrane [39], followed by coumarin-based inhibitors of bacteria replicative DNA helicase [40]. Similar to bacterial activity, among the four compounds, herniarin showed the most potential anticancer activity against four tested cancer cell lines. Although we accessed the activity of herniarin and skimmin against AGS cell line for the first time, these compounds could be used in further studies to reveal their exact mechanisms of action and their anti-cancer effects in vivo. The previous study has reported that umbelliferone have growth inhibitory effects on human cancer cell lines such as breast cancer (MCF-7) and lung cancer (H727) [41]. Esculetin did not show activity against the tested cell lines in this study. However, it has anti-proliferative effects on G361 human malignant melanoma [4] and U937 human leukemia cells [42] and. These results provide the ability that modification of umbelliferone might generate a promising anticancer agent against various cancer cell lines.

## Conclusion

In conclusion, the results demonstrate that both native enzyme and engineered enzyme expressed in the whole cell allow producing the precious compounds. As a result we successfully produced three different molecules herniarin, esculetin and skimmin from umbelliferone using engineered microbial cells. The employed fermentation approach is cheaper than in vitro reaction and other chemical synthesis methods. The biosynthesized molecules were also accessed for their potential bioactivities against different pathogens and cancer cell lines. The present research suggests that the combinatorial biosynthesis of different biosynthetic enzymes could rapidly promote to a novel secondary metabolite.

## Methods

### Chemicals and reagents

Standard umbelliferone, *δ*–aminolevulinic acid hydrochloride (*δ*–ALA), SAM, deuterium oxide (D_2_O), and DMSO-d_6_ were purchased from Sigma-Aldrich (USA). UDP-*α*-D-glucose, *α*-D-glucose 1-phosphate, and isopropyl-*β*-D-thiogalactopyranoside (IPTG) were obtained from Genechem (Korea). NADP oxidized form was provided from Tokyo Chemical Industry Co., Ltd (Japan). HPLC-grade acetonitrile and water were purchased from Mallinckrodt Baker (Phillipsburg, NJ, USA). All other chemicals used were of analytical grade.

### Plasmids, microorganisms, and culture conditions

Plasmid pCW(Ori^+^)-mutant 13 (M13) carrying amino acid substitution relative to wild type CYP450 BM3: M13 (R47L/L86I/F87V/L188Q) [43] and pET28a-YjiC were constructed previously [24]. The sequence of previously characterized *S. avermitilis* derived OMT [29] was cloned using the first cloning site of pRSF-Duet vector to make recombinant plasmid pRSF-Duet-SaOMT2. These plasmids were transformed into *E. coli* BL21 (DE3) (Stratagene, USA) using standard procedures. 100 μg/mL of ampicillin and 50 μg/mL of kanamycin were used in LB medium for protein expression and biotransformation assay.

### Enzyme expression and purification

*E. coli* BL21 (DE3) harboring recombinant pCW(Ori^+^)-P450 BM3 with pCW(Ori^+^)-mutant 13, pET28a-YjiC, and pRSF-Duet-SaOMT2 were cultured in LB broth containing appropriate antibiotics at 37 °C and 180 rpm for 6 h. 0.5 mM IPTG and 1 mM *δ*–ALA were used to expression of CYP450 BM3 and mutant 13 when cell optical density at 600 nm (OD_600__nm_) reached 0.6. These cells were further incubated for an additional 20 h at 28 °C before harvest. Furthermore, 0.5 mM IPTG was used to induced for protein expression of pET28a-YjiC and pRSF-Duet-SaOMT2 at 20 °C for 20 h. These cells were harvested by centrifugation at 842 × *g* for 10 min and suspended twice in 100 mM Tris–HCl (pH 7.6) buffer containing 10% glycerol. Cells were suspended in 1 mL of the same buffer and lysed by sonication using a Sonosmasher (Ultrasonic, Inc.). Following centrifugation at 13,475 × *g* for 30 min at 4 °C, the protein fractions were applied across 12% sodium dodecyl sulfate polyacrylamide gel electrophoresis (SDS-PAGE). Amicon®Ultra-0.5 mL 100 K and 30 K devices (Merck, USA) were used to collect soluble cytosolic fraction of CYP450 BM3, mutant 13, and YjiC, SaOMT2, respectively. 100 mM Tris–HCl (pH 7.6) buffer containing 10% glycerol was used to store solution fractions at −20 °C until next use. CYP450 BM3 protein content (nmol) = [(absorbance difference × 1000)/91 mM^−1^cm^−1^] x dilution factor [44]. Protein concentration was determined via Bradford method [45].

### Enzyme essay

The reaction mixture was performed in 200 μL with 1 mM substrate. Hydroxylation reaction contained 50 μg/mL CYP450 BM3 or mutant 13, 100 mM potassium phosphate buffer (pH 7.6), and 10 mM MgCl_2_.6H_2_O. The sample was pre-incubated at 37 °C for 15 min and the reaction was initiated by the addition of NADPH regenerating system consisting of 10 mM glucose-6-phosphate, 0.5 U glucose-6-phosphate-dehydrogenase, and 0.5 mM NADP^+^. Glucosylation reaction mixture was incubated with 2 mM UDP-*α*-D-glucose, 10 mM MgCl_2_, 50 μg/mL purified YjiC in 100 mM Tris–HCl buffer (pH 7.6). Methylation reaction contained 50 μg/mL of purified SaOMT2 and 10 mM MgSO_4_ with 2 mM SAM as methyl donor in 100 mM Tris–HCl buffer (pH 7.6). Reactions were incubated at 37 °C for 30 min. These reactions were stopped by adding chilled methanol at twice volume followed by vigorous shaking for 15 min. Mixtures were then centrifuged at 13,475 *x g* for 30 min. The supernatants were used to HPLC-PDA and HR-QTOF ESI/MS. The conversion percentage of each substrate was calculated through intergrated between substrate and product peak area. A calibration standard was created using different concentration of umbelliferone (10, 25, 50, 100, and 200 μg/mL).

### Whole cell biotransformation

Seed cultures of *E. coli* BL21 (DE3) harboring recombinant plasmids were prepared in 6 mL LB broth with appropriate antibiotics followed by incubation at 37 °C with shaking at 180 rpm for approximately 6 h. For whole cell reaction of CYP450 mutant 13, 200 μL of pre-inoculum was transferred into 250 ml flask containing 50 mL of different media (LB, TB, 1x M9 minimal salt) with appropriate antibiotic and incubated at 37 °C. When OD_600_ reached 0.8, the culture was induced with 1.0 mM IPTG and 0.5 mM *δ*-ALA and incubated at 28 °C for 12 h. Biotransformation of *E.coli* harboring pET28a-YjiC and pRSF-Duet-SaOMT2 was induced by 0.5 mM IPTG at 20 °C for 12 h. 100 mM umbelliferone was supplemented to the same samples. The culture was incubated for an additional 12 h, collected, extracted and analyzed.

### Scale-up of whole cell biocatalyst system in a fermentor

Fermentation of three *E.coli* BL21 (DE3) recombinant strains (CYP450 mutant 13, YjiC, and SaOMT2) was carried out in 3-L media under optimal conditions as described previously [34, 46]. The quantify of hydroxylated and methylated production were taken every 12 h until 48 h. Likewise, glycosylated sample was taken every 3 h up to 15 h. Then, culture broth was centrifuged. Finally, the supernatant was extracted with twice volume of EtOAc.

### Analytical methods

The culture broth was extract with double volume of EtOAc (v/v = 2:1) using Soxhlet extractor. Soxhlet extractor was then kept still to separate two layers after the mixture was shaken for 12 h at room temperature. A rotary evaporator was used to dried EtOAc fraction. The products were analyzed by HPLC-PDA using a reversed-phase column (Mightysil RP–18 GP 250–4.6 (5 μm), Kanto Chemical, Japan) at 330 nm. The binary mobile phases were include solvent A [0.05% trifluroacetic acid in HPLC-grade water] and solvent B (100% acetonitrile). The total flow rate was kept at 1 mL/min for 30 min. The percentages of solvent B used were as follows: 0–15% (0–4 min), 45% (4–10 min), 75% (10–14 min), 90% (14–20 min), 10% (20–25 min), 10% (25–30 min). The compounds were purified by preparative HPLC (Shimazu, Tokyo, Japan) with C_18_ column (YMC–Pack ODS-AQ (250 × 20 mm I.D., 10 μm) linked to a UV detector (330 nm). HR–QTOF ESI/MS analysis using an ACQUITY UPLC® coupled with SYNAPT G2-S (Water Corp., USA). For NMR analysis of the purified product, compounds were dried, lyophilized, and dissolved in DMSO-d_6_ and subjected to 700 MHz Bruker Biospin NMR for one-dimensional ^1^H-NMR, ^13^C-NMR, and two-dimensional HMBC analyses.

### Solubility study

50 μM of umbelliferone, esculetin, skimmin and herniarin were separately dissolved in 200 μL phosphate-buffered saline (PBS) at pH 7.6. The mixtures were vortexed for 30 min and centrifuged at 13,475 *x g* for 15 min. The supernatants were collected and filtered through 0.45-μm syringe filters. Subsequently, aliquots (20 μL) were analyzed by HPLC-PDA at 330 nm. The concentrations of all compounds dissolved in PBS were determined by regression equations.

### Antibacterial activity

Three Gram-positive bacteria (*Staphylococcus aureus* subsp. *aureus* KCTC 1916, *Bacillus subtilis* KACC 17047, and *Micrococcus luteus* KACC 13377) and two Gram-negative bacteria (*Pseudomonas aeruginosa* KACC 10232 and *Enterobacter cloaceae* subsp. *disolvens* KACC 13002) were used to test antibacterial activity of umbelliferone and its derivatives. The paper disc diffusion assay on Mueller-Hinton agar (MHA) plate were carried out. Inocula containing 10^7^ colony forming units (CFU)/mL were spread onto MHA plates. 10 μL of 100 mM compounds were placed on the surface of inoculated agar plates through sterile filter paper discs. The samples were then incubated at 37 °C for 12 h. The zone of inhibition diameter was measured in millimeter [47].

### Anticancer activities

Four cencer cell lines (B16F10, AGS, HeLa, and HepG2) grown in Roswell Park Memorial Institute 1640 medium containing 10% fetal bovine serum (FBS) (Invitrogen, USA) were applied to test anticancer activities of umbelliferone and its derivatives. All cells were maintained at 37 °C in a humidified 5% CO_2_ incubator. For cell growth assay, cells seeded at 2 x 10^3^ cell/well in 96-well plates (SPL Life Sciences, Korea) were treated with each compound after serial dilution (400 μM, 200 μM, 100 μM, 50 μM, 25 μM, 12.5 μM) for 72 h. Cell viability was measured using MTT colorimetric assay [48].

### Statistical analysis

Values are mean ± standard deviation (SD). SD was calculated from the results of three independent experiments. Differences with *p* value < 0.05 were indicated a statistically significant.

## Additional files

Additional file 1: Table S1. The disc-diffusion assay showed the inhibition zone diameter (mm) of four compounds against various Gram-positive and Gram-negative bacteria. **Table S2**. IC_50_ values of four compounds against B16F10, AGS, HeLa and HepG2 cell lines. **Figure S1**. SDS-PAGE analysis of four recombinant proteins used in the study. **Figure S2**. The UV maxima absorbance and exact mass analysis of umbelliferone (A) and reaction products P1 (B), P2 (C), P3 (D). **Figure S3**. Hydroxylated umbelliferone production optimization and cell growth. **Figure S4**. Glycosylated umbelliferone production optimization and cell growth. **Figure S5**. Methylated umbelliferone production optimization and cell growth. **Figure S6**. 1-Dimensional NMR of umbelliferone standard. **Figure S7**. 1-Dimensional NMR of esculetin. **Figure S8**. NMR of skimmin. **Figure S9**. NMR of herniarin. (DOCX 27595 kb)

## Abbreviations

AGS: Gastric carcinoma
B16F10: Skin melanoma
DMSO: Dimethyl sulfoxide
HeLa: Epitheliod cervix carcinoma
HepG2: Hepatic carcinoma
HPLC-PDA: High performance liquid chromatography‑photodiode array
HR-QTOF ESI/MS: High resolution-quadruple time of flight electrospray ionization/mass spectrometry
IPTG: Isopropyl-*β*-D-thiogalactopyranoside
NADP^+^: *β*-Nicotinamide adenine dinucleotide phosphate oxidized
NADPH: *β*-Nicotinamide adenine dinucleotide phosphate reduced
NMR: Nuclear magnetic resonance
*δ*-ALA: *δ*-aminolevulinic acid hydrochloride
