# Supplementary material for: Synthesis of umbelliferone derivatives in Escherichia coli and their biological activities
Source: J Biol Eng. 2017 Apr 5;11:15. doi: 10.1186/s13036-017-0056-5 (PMC5382406; doi:10.1186/s13036-017-0056-5)

Additional files for:

***Journal of Biological Engineering***

Synthesis of umbelliferone derivatives in *Escherichia coli* and their biological activities

Luan Luong Chu

[chuluongluan218@gmail.com](mailto:chuluongluan218@gmail.com)

Ramesh Prasad Pandey

[pandey@sunmoon.ac.kr](mailto:pandey@sunmoon.ac.kr)

Haet Nim Lim

[lhn92@naver.com](mailto:lhn92@naver.com)

Hye Jin Jung

[poka96@sunmoon.ac.kr](mailto:poka96@sunmoon.ac.kr)

Nguyen Huy Thuan

[thuanbiochem@gmail.com](mailto:thuanbiochem@gmail.com)

Taesu Kim

taesuda@sunmoon.ac.kr

Jae Kyung Sohng*

[sohng@sunmoon.ac.kr](mailto:sohng@sunmoon.ac.kr)

***Corresponding author**: *Department of BT-Convergent Pharmaceutical Engineering,* *Sun Moon University, 70 Sunmoon-ro 221, Tangjeong-myeon, Asan-si, Chungnam 336-708, Republic of Korea.*

Tel: +82(41)530-2246 Fax: +82(41)544-2919

**Table of contents**

**Table S1.** The disc-diffusion assay showed the inhibition zone diameter (mm) of four compounds against five different human pathogens.

**Table S2.** IC_50_ values of four compounds against B16F10, AGS, HeLa and HepG2 cell lines.

**Figure S1**. SDS-PAGE analysis of four recombinant proteins used in the study. *Lane M,* standard protein molecular mass marker; *lane 1*, soluble protein fraction; *lane 2*, insoluble protein fraction. (**A**) cell lysate containing CYP450 BM3 (119 kDal). (**B**) cell lysate containing M13 (119 kDal). **(C**) cell lysate containing YjiC (45 kDal). (**D**) cell lysate containing SaOMT2 (37.5 kDal).

**Figure S2.** The UV maxima absorbance and found mass analysis of umbelliferone (**A**) and reaction products P1 (**B**), P2 (**C**), P3 (**D**). P1 have been identified as hydroxylated umbelliferone while P2 and P3 have been identified as glycosylated and methylated umbelliferone, respectively.

**Figure S3.** Hydroxylated umbelliferone production optimization and cell growth. (**A)** Differences between CYP450 BM3 and M13 on hydroxylated umbelliferone production under various medium consist of 2% glucose and 100 µM substrate at 48 h incubation. (**B)** The various concentration of umbelliferone (100-1000 µM) and (**C**) corresponding to cell growth at OD_600_ nm with M13 on M9 medium.

**Figure S4**. Glycosylated umbelliferone production optimization and cell growth. (**A**) Differences in glycosylated umbelliferone production between LB, TB and M9 medium supplied 2% glucose and 100 µM substrate at 12 h incubation. (**B)** The various concentration of umbelliferone (100-2000 µM) and (**C**) corresponding to cell growth at OD_600_ nm with Yjic on M9 medium.

**Figure S5**. Methylated umbelliferone production optimization and cell growth. (**A**) Differences in methylated umbelliferone production between LB, TB and M9 medium supplied 2% glucose and 100 µM substrate at 48 h incubation. (**B)** The various concentration of umbelliferone (100-500 µM) and (**C**) corresponding to cell growth at OD_600_ nm with SaOMT2 on LB medium.

**Figure S6.** 1-Dimensional NMR of umbelliferone standard. (**A**) ^1^H-NMR, (**B**) ^13^C-NMR.

**Figure S7.** 1-Dimensional NMR of esculetin. (**A**) ^1^H-NMR, (**B**) ^13^C-NMR.

**Figure S8.** NMR of skimmin. (**A**) ^1^H-NMR, (**B**) ^13^C-NMR, (**C**) HMBC close view of sugar region

**Figure S9.** NMR of herniarin. (**A**) ^1^H-NMR, (**B**) ^13^C-NMR, (**C**) HMBC close view of methyl region

**Table S1**

| **Pathogens** | *B. subtilis* | *M. luteus* | *S. aureus* | *P. aeruginosa* | *E. cloaceae* |
| --- | --- | --- | --- | --- | --- |
| Umbelliferone | **–** | **–** | **–** | **–** | **–** |
| Esculetin | **–** | **–** | **–** | **–** | **–** |
| Skimmin | **–** | **–** | **–** | **–** | **–** |
| Herniarin | 8.5 ± 0.18 | **–** | 9 ± 0.12 | **–** | **–** |
| *(*–*)* denotes no inhibition zone | | | | | |

**Table S2**

| **IC_50_ (μM)** | Umbelliferone | Esculetin | Skimmin | Herniarin |
| --- | --- | --- | --- | --- |
| B16F10 | 328.9 | > 400 | > 400 | 197.0 |
| AGS | 129.9 | > 400 | 34.42 | 2.829 |
| HeLa | > 400 | 340.8 | 375.4 | 80.21 |
| HepG2 | 222.3 | > 400 | > 400 | 206.1 |

**Figure S1**

**
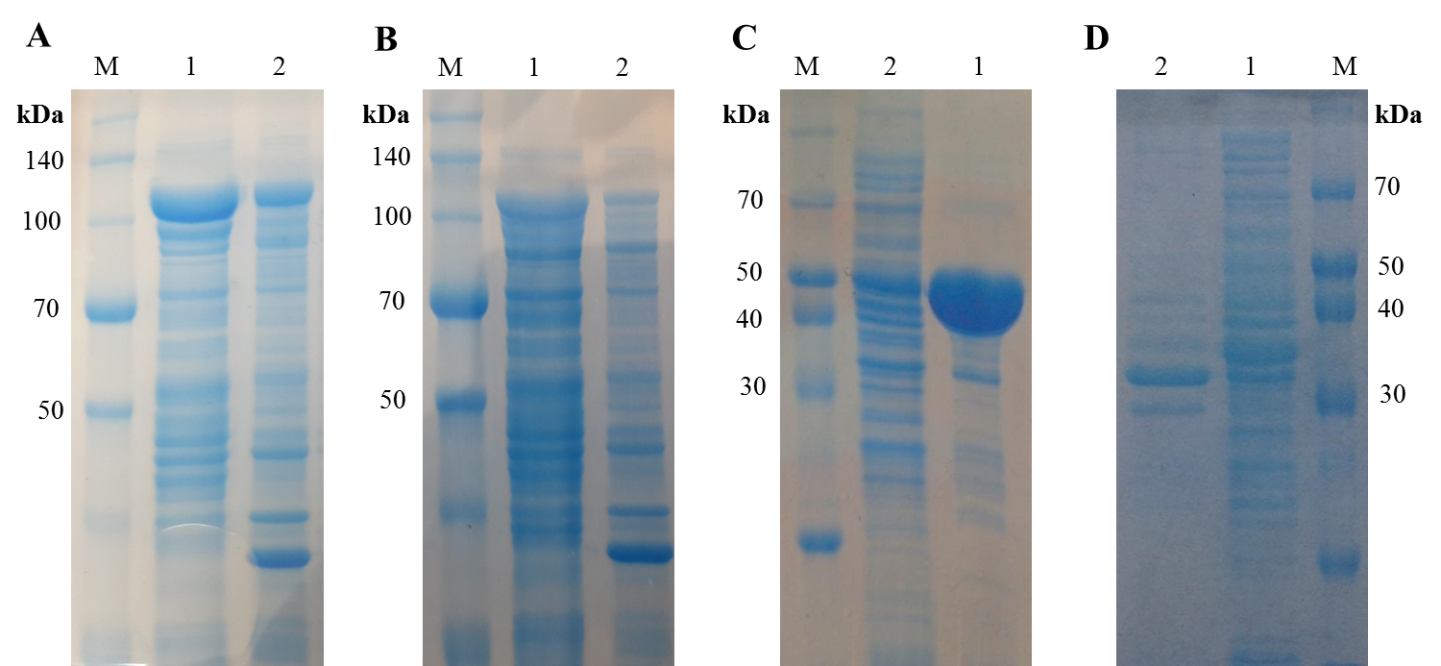
**

**Figure S2**

**
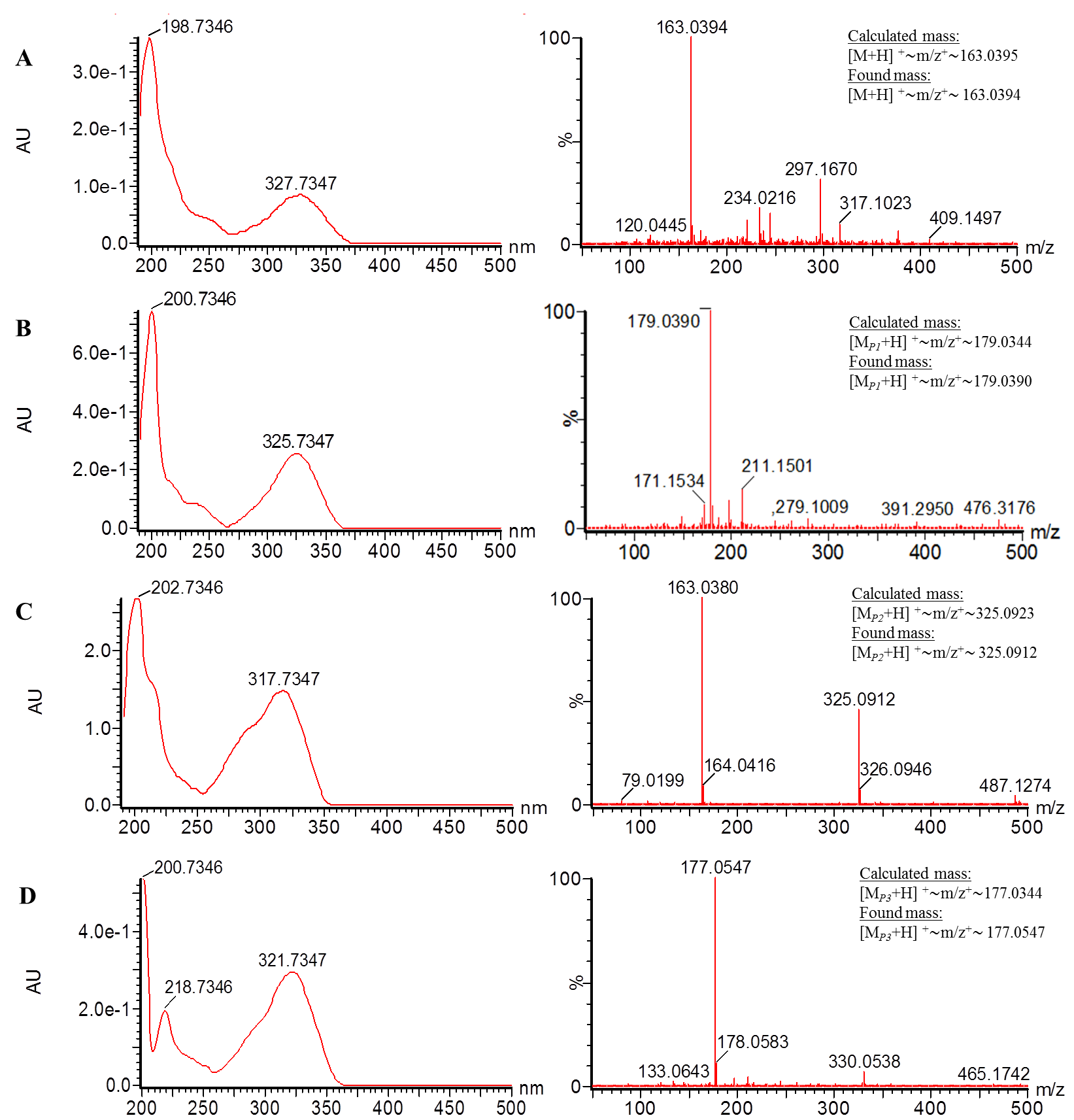
**

**Figure S3**

**
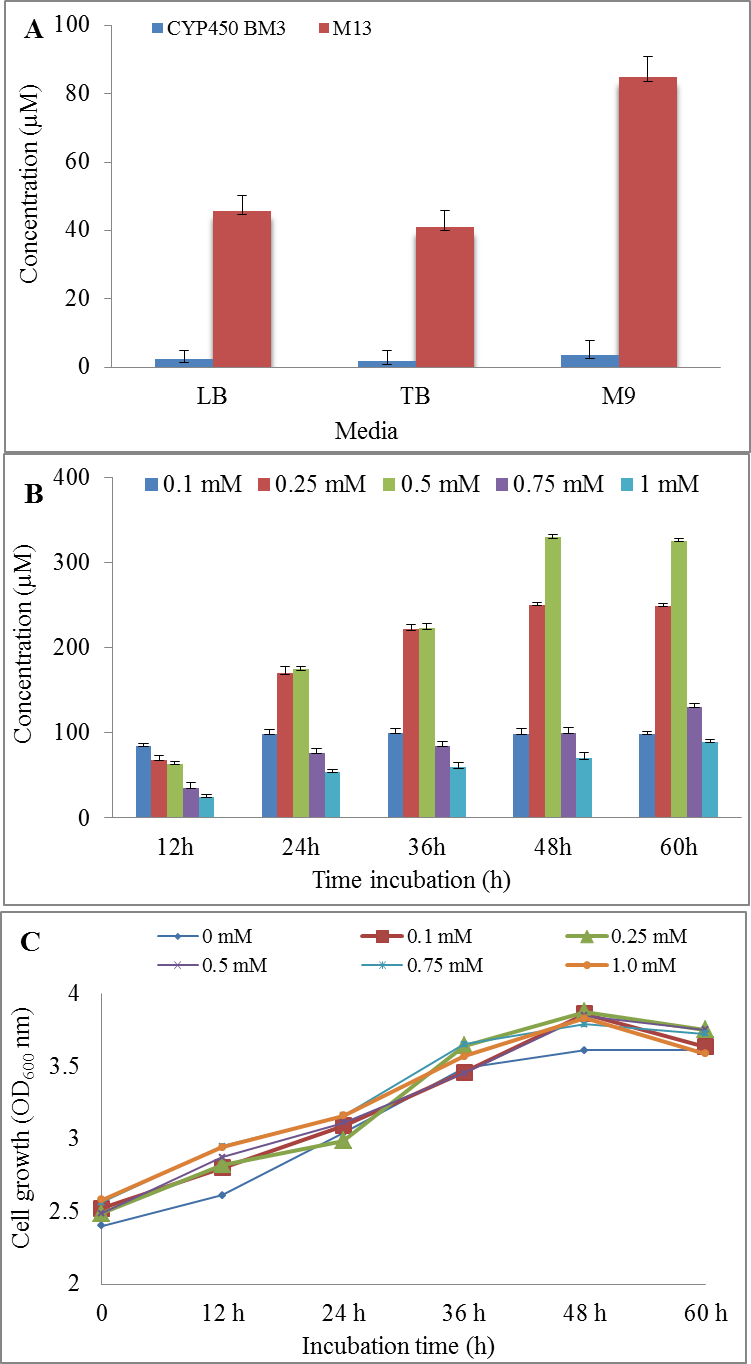
**

**Figure S4**

**
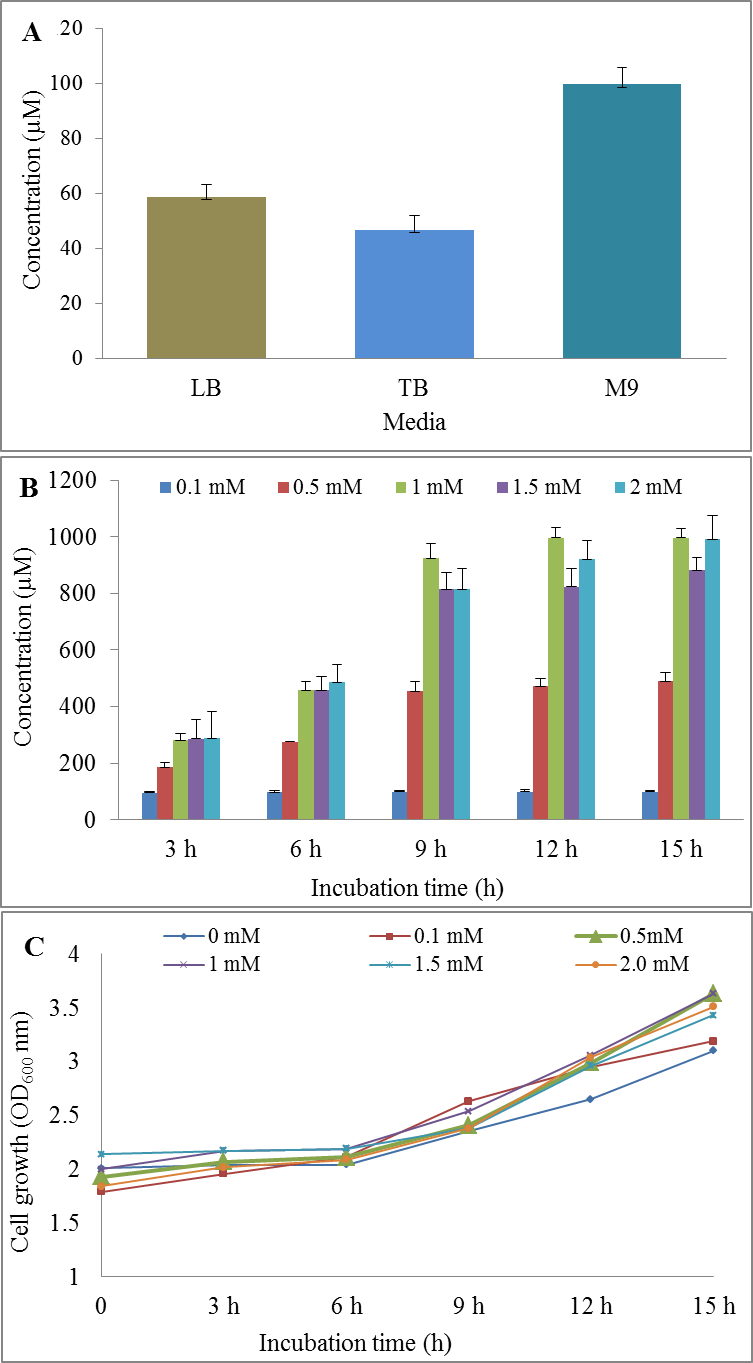
**

**Figure S5**

**
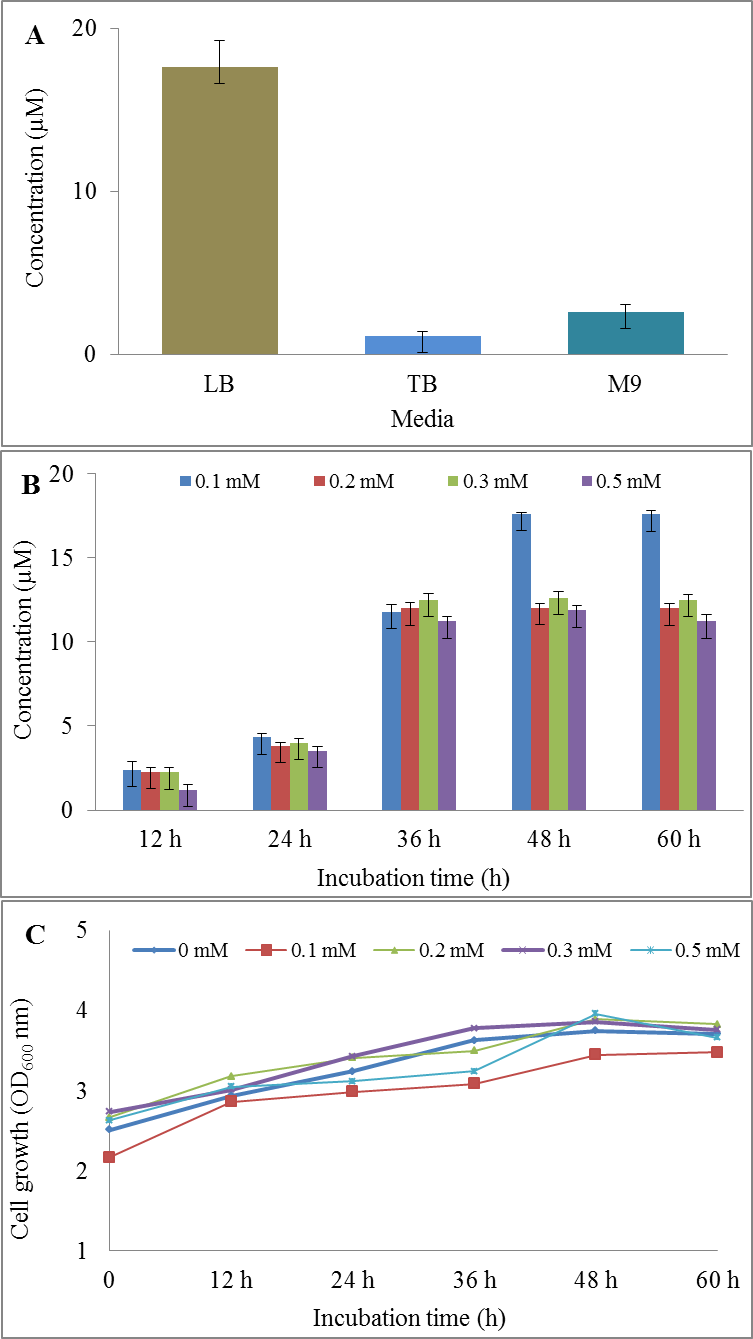
**

**Figure S6.** 1-Dimensional NMR of umbelliferone standard

1. ^1^H-NMR


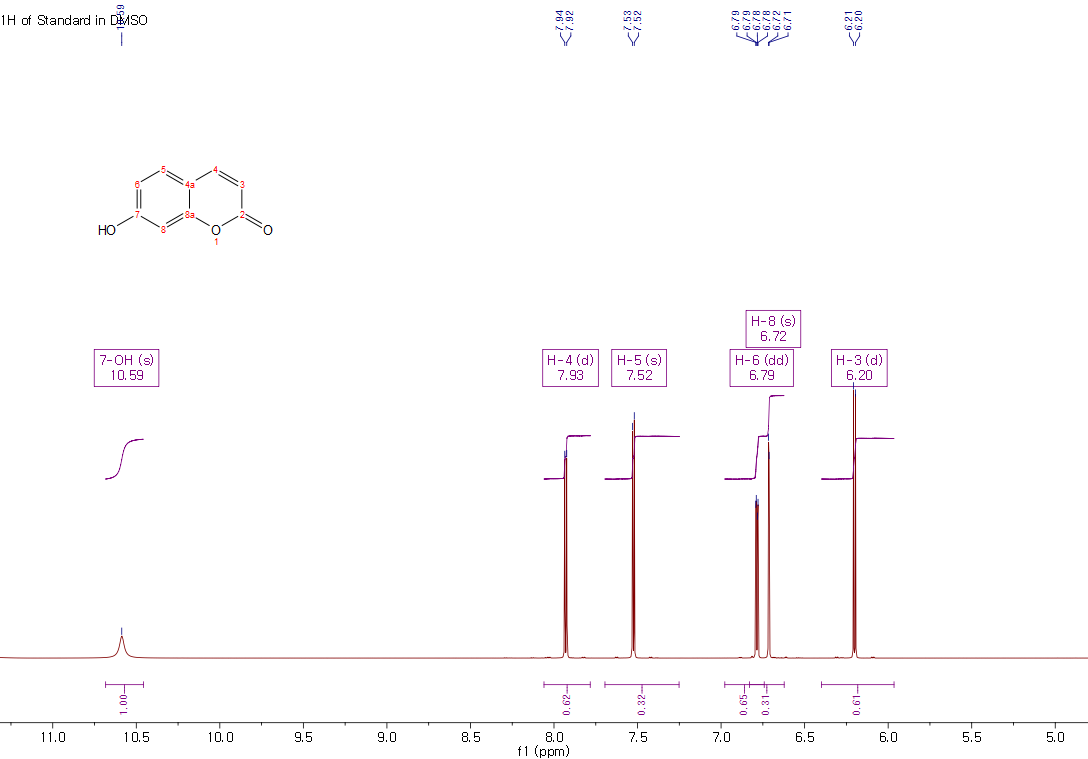


1. ^13^C-NMR.


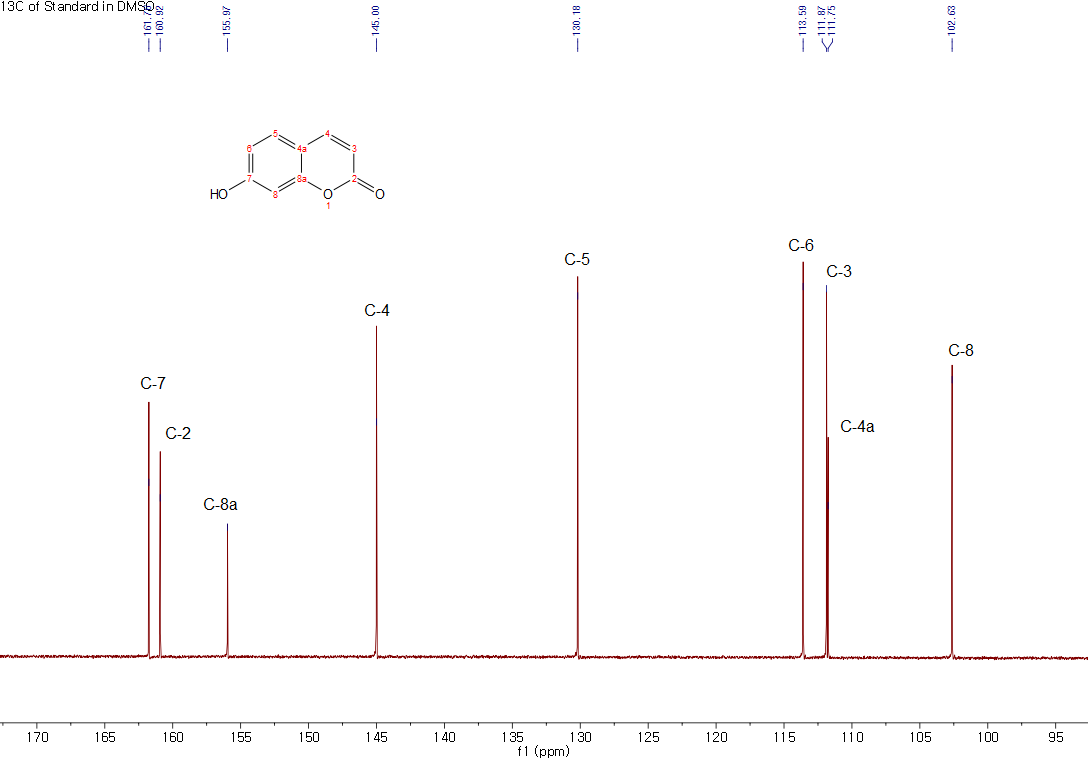


**Figure S7.** 1-Dimensional NMR of esculetin

1. ^1^H-NMR


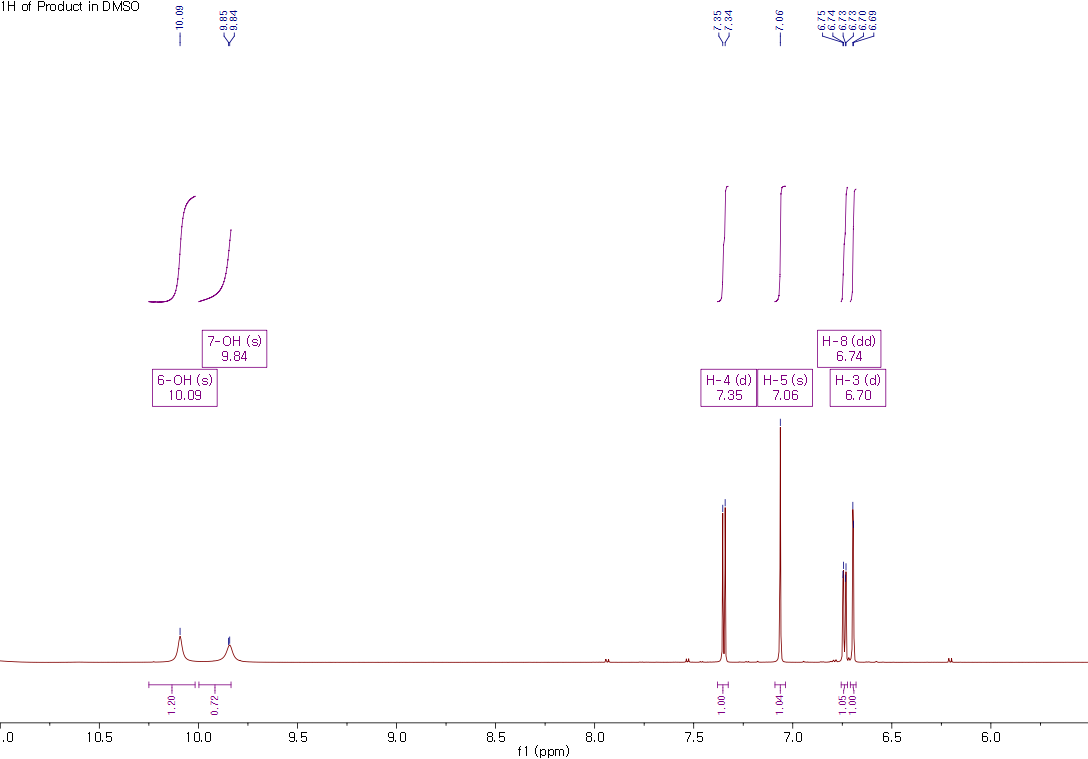


1. ^13^C-NMR


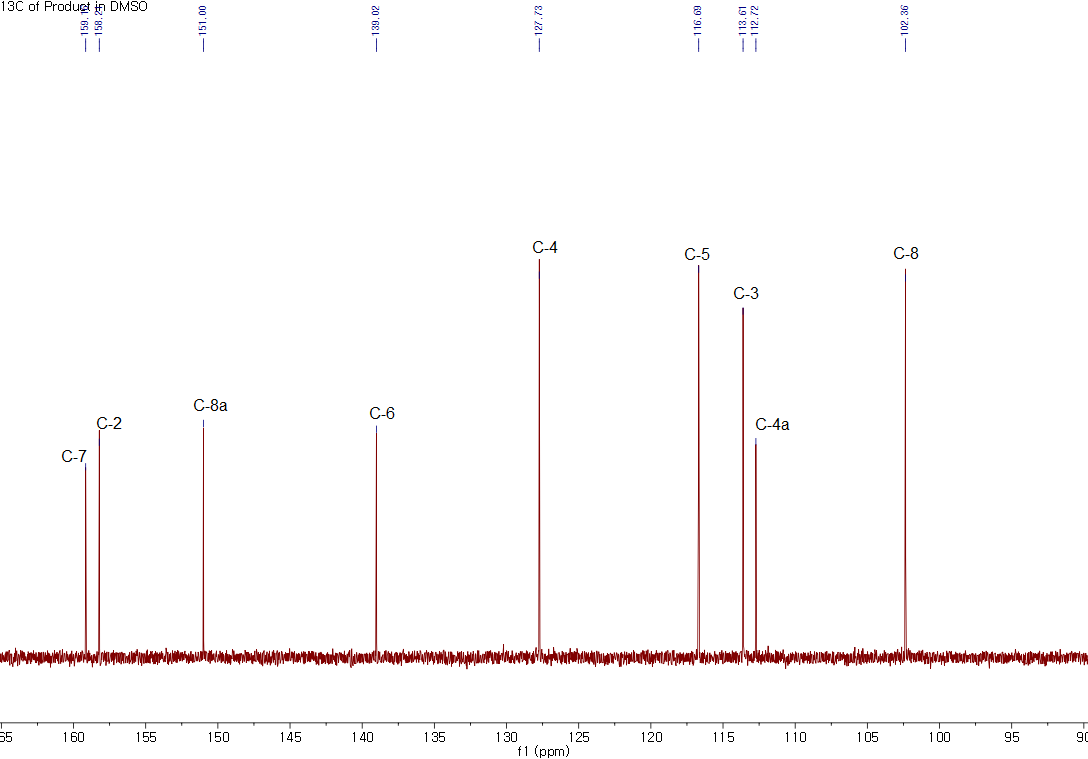


**Figure S8.** NMR of skimmin

1. ^1^H-NMR


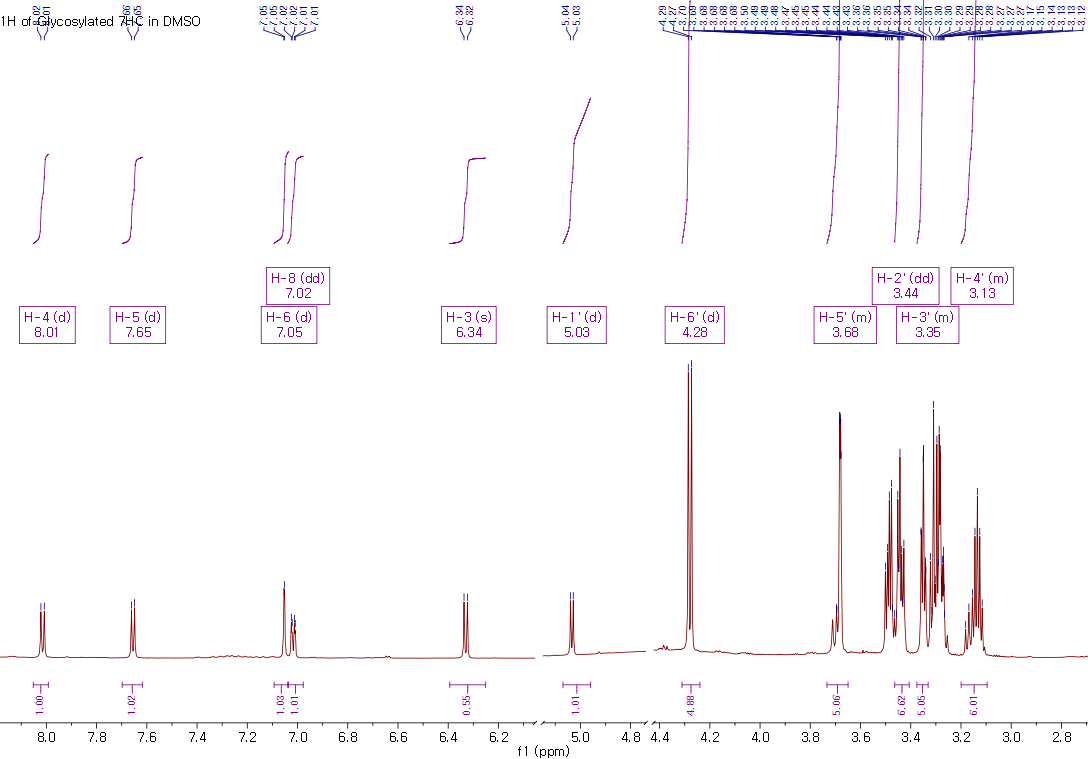


1. ^13^C-NMR


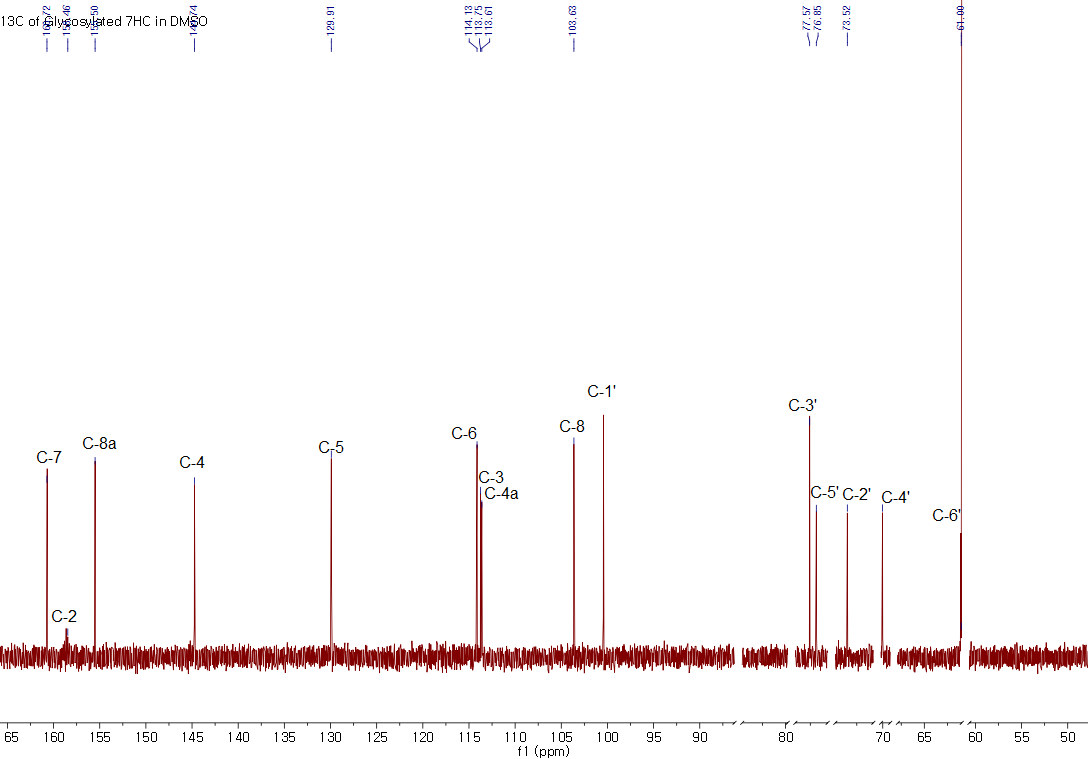


1. HMBC close view of sugar region


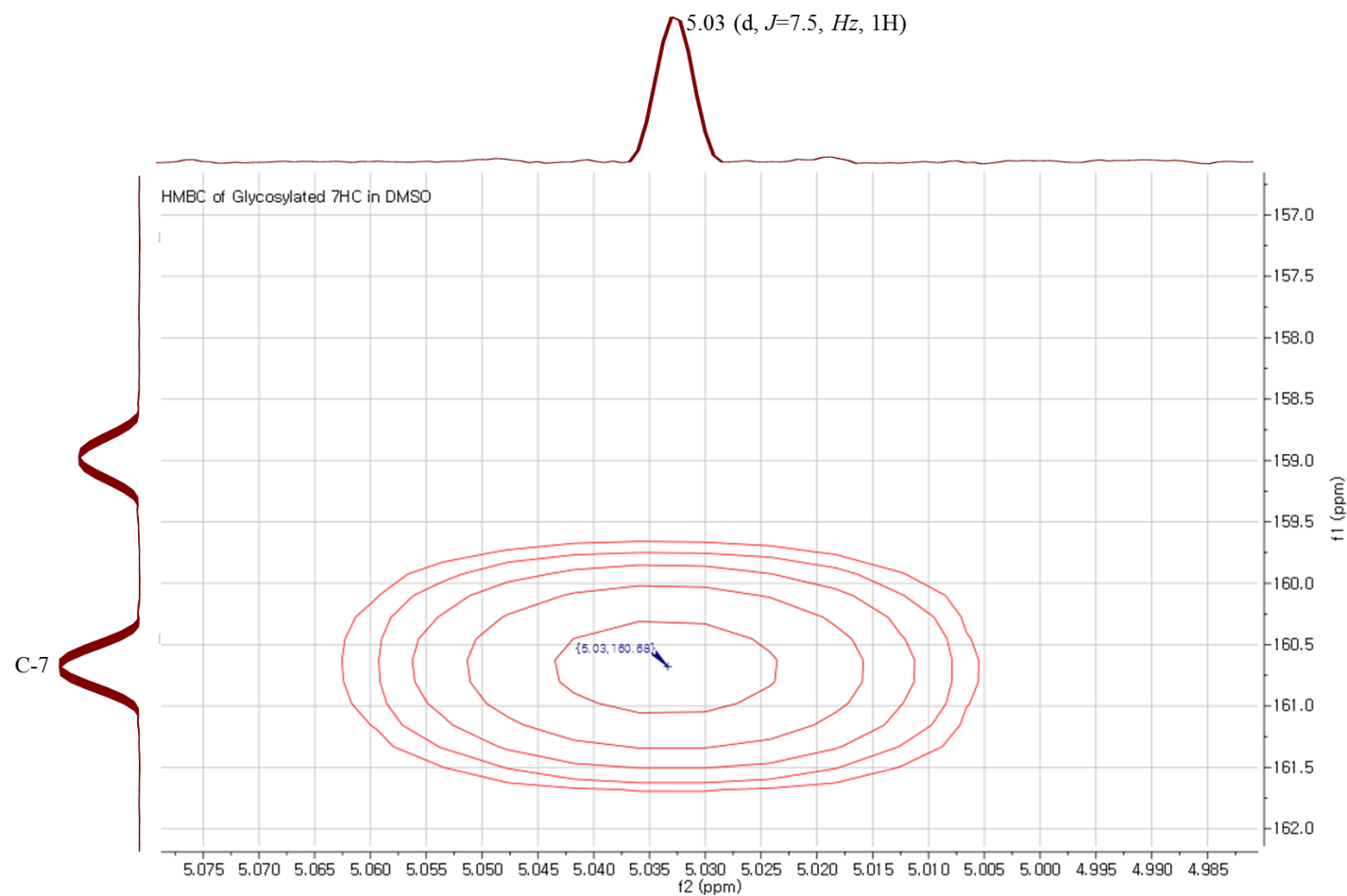


**Figure S9.** NMR of herniarin

1. ^1^H-NMR


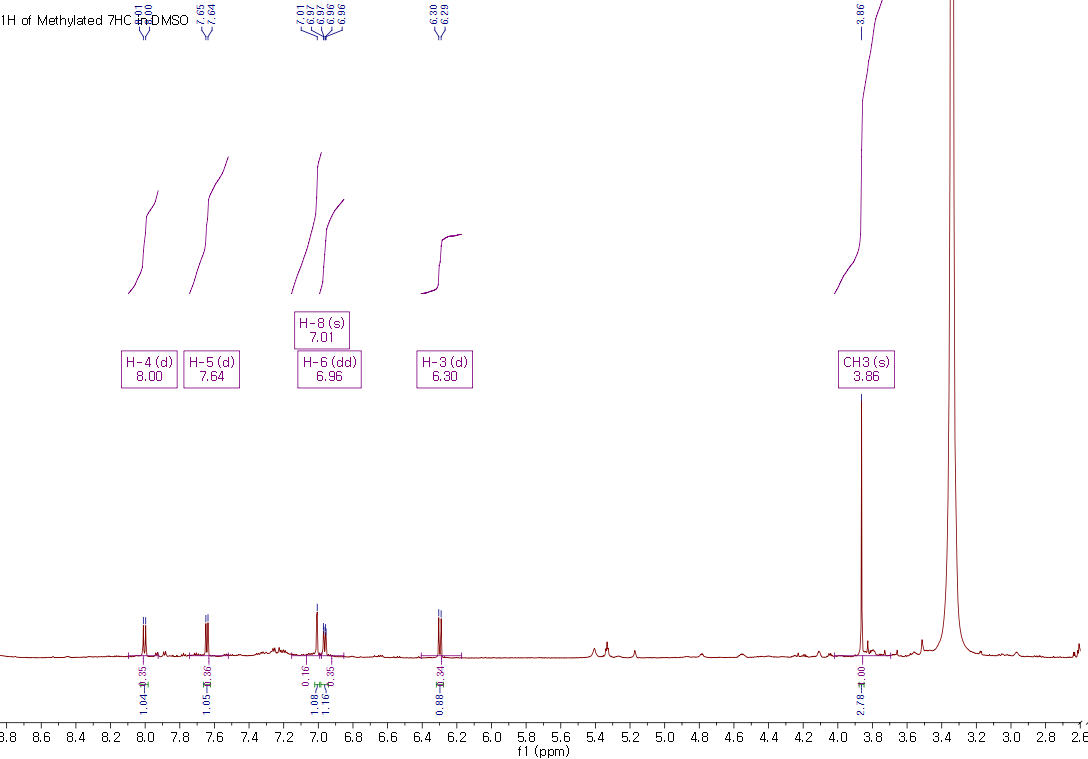


1. ^13^C-NMR


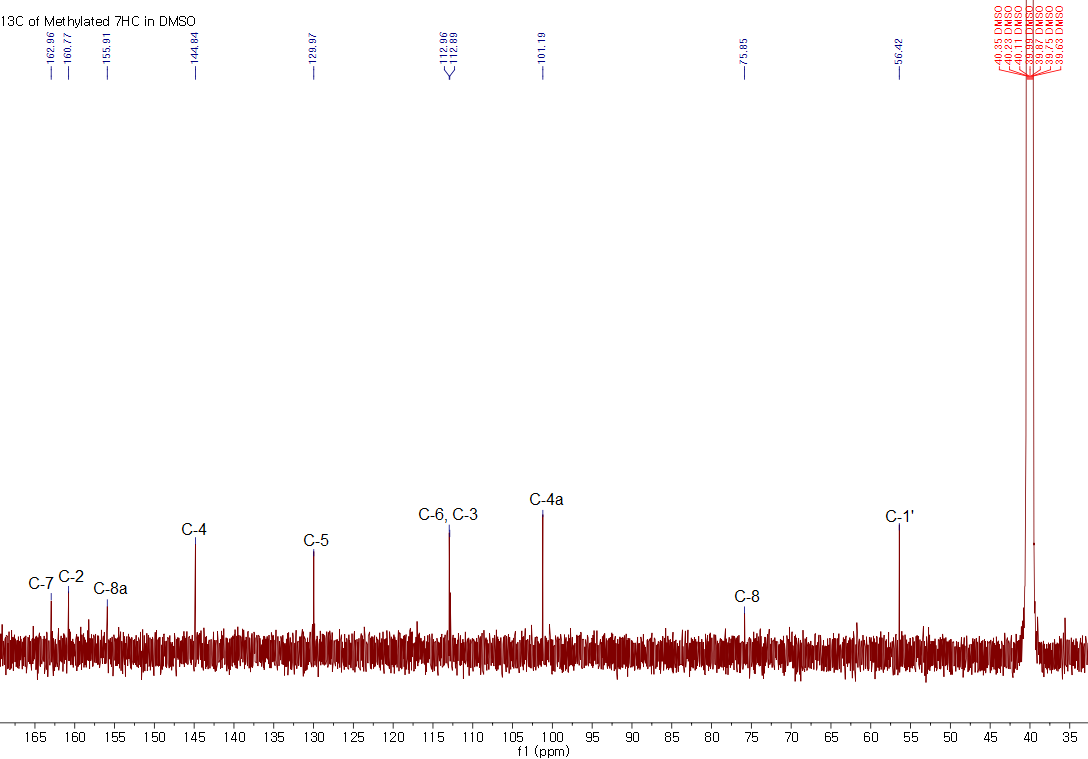


1. HMBC close view of methyl region


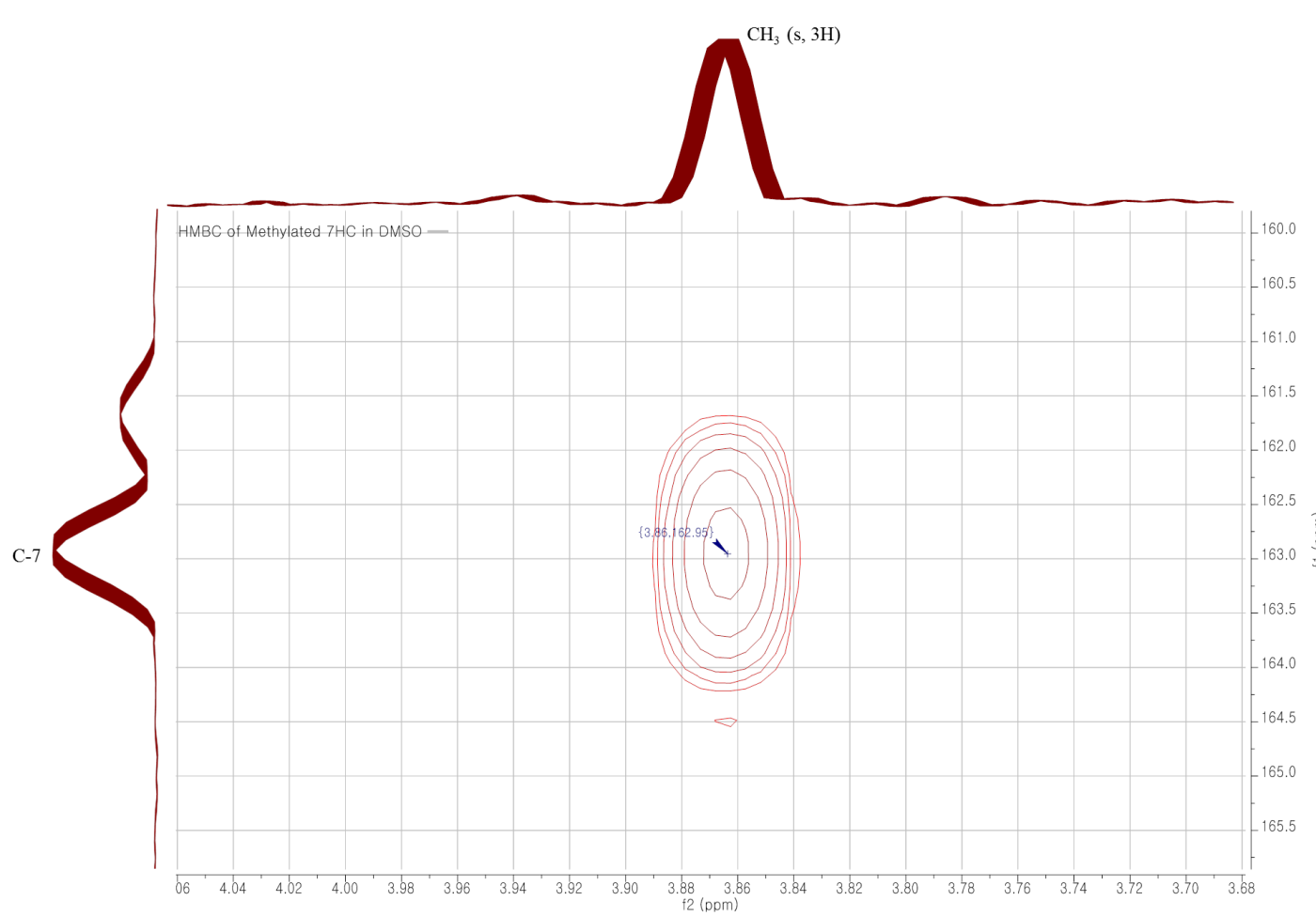

Supplement: Supplementary file 1 — The disc-diffusion assay showed the inhibition zone diameter (mm) of four compounds against various Gram-positive and Gram-negative bacteria. Table S2. IC50 values of four compounds against B16F10, AGS, HeLa and HepG2 cell lines. Figure S1. SDS-PAGE analysis of four recombinant proteins used in the study. Figure S2. The UV maxima absorbance and exact mass analysis of umbelliferone (A) and reaction products P1 (B), P2 (C), P3 (D). Figure S3. Hydroxylated umbelliferone production optimization and cell growth. Figure S4. Glycosylated umbelliferone production optimization and cell growth. Figure S5. Methylated umbelliferone production optimization and cell growth. Figure S6. 1-Dimensional NMR of umbelliferone standard. Figure S7. 1-Dimensional NMR of esculetin. Figure S8. NMR of skimmin. Figure S9. NMR of herniarin. (DOCX 27595 kb) [file 13036_2017_56_MOESM1_ESM.docx]
